# Imaging human coronary cholesterol/urate crystals with cross-polarized micro-optical coherence tomography

**DOI:** 10.3389/fcvm.2024.1433227

**Published:** 2024-10-28

**Authors:** Kensuke Nishimiya, Gargi Sharma, Kanwarpal Singh, Osman O. Ahsen, Joseph A. Gardecki, Guillermo J. Tearney

**Affiliations:** ^1^ Wellman Center for Photomedicine, Harvard Medical School and Massachusetts General Hospital, Boston, MA, United States; ^2^ Department of Cardiovascular Medicine, Tohoku University Graduate School of Medicine, Sendai, Japan; ^3^ Harvard-MIT Division of Health Sciences and Technology, Massachusetts Institute of Technology, Cambridge, MA, United States; ^4^ Department of Pathology, Harvard Medical School Mass General Brigham, Boston, MA, United States

**Keywords:** optical coherence tomography, coronary artery disease, cholesterol crystals, gout, uric acid, inflammation, cross-polarization

## Abstract

**Introduction:**

Birefringent crystals such as monosodium-urate (MSU) and cholesterol crystals (CC) likely contribute to the progression of coronary artery disease (CAD) due to their potential to exacerbate inflammation through inflammatory cytokine activation. Here, we present cross-polarized micro-optical coherence tomography (CP-µOCT) for visualizing individual birefringent crystals in human coronary arteries.

**Methods and results:**

Human cadaver coronary arteries with a history of CAD with or without gout were dissected for CP-µOCT imaging. Specimens were processed for histological identification of birefringence under polarization light microscopy (PLM). CP-µOCT visualized needle-crystals that appeared as long projections in orthogonal planes, and PLM confirmed that CP-µOCT-delineated needle-crystals demonstrated negative birefringence. The needle-crystals were dissolved after immersion in uricase (*p* < 0.05), and thus were MSU. CP-µOCT was three-dimensionally volume-rendered for counting MSU and CCs in 79 regions of interest sized [750 (*x*) × 500 (*y*) × 400 (*z*) µm]. Crystal counts were normalized by the total coronary length utilized. The relationship between CP-µOCT-delineated MSU counts and those seen in corresponding histology, and the difference in coronary MSU amongst gout vs. non-gout patients was analyzed. CP-µOCT-delineated MSU counts were significantly correlated with MSU counted by PLM-based histology (*R* = 0.98, *p* < 0.01), and with histology-derived intimal thickening (*R* = 0.51, *p* < 0.01). MSU and CCs were both significantly greater in gout patients compared with non-gout patients (*p* < 0.05).

**Discussion:**

These results demonstrate a significant increase in CP-µOCT-delineated crystals in gout vs. non-gout patients, suggesting that this technology can be used to improve our understanding of crystal-driven coronary pathogenesis.

## 1 Introduction

Birefringent crystalline components, such as cholesterol crystals (CCs), are commonly noted in coronary atherosclerotic plaques (1). CCs have long been identified as a therapeutic target for cardiovascular disease, due to the potential of these crystals to exacerbate inflammation through NLRP3 (NOD, LRR and pyrin domain containing protein 3) inflammasome-mediated cytokine production/activation (2). Monosodium urate (MSU) crystals are tiny needle-shaped crystals (3) that are commonly observed in synovial fluid of gout patients (4, 5). Similarly to CCs, MSU has shown to induce gouty inflammation through NLRP3 inflammasome activation, and interleukin-1β secretion (6). Basic studies demonstrated that colchicine, which exerts anti-inflammatory effects on gouty flares, can inhibit the NLRP3 inflammasome that can be triggered by crystals (7). Indeed, colchicine has shown to reduce the risk of cardiovascular events after acute myocardial infarction (8) and stable coronary artery disease (CAD) (9), highlighting the possible clinical significance of therapies to curb vascular inflammation.

We have recently developed a 1-µm-resolution-optical coherence tomography (µOCT) technology that enables the visualization of cellular and sub-cellular structures in human coronary arteries (10–13). The feasibility of µOCT for visualization of CCs in human cadaver coronary arteries has been reported (10–12). Yet, standard µOCT has difficulty reliably quantifying MSU because of the small size of the single crystals and high background signal from surrounding tissue. Cross-polarized OCT was developed to visualize birefringent components by increasing the OCT image contrast (14, 15). In this work, we introduce cross-polarized (CP)-µOCT, which combines the enhanced contrast of CP-OCT imaging with the high resolution of µOCT. We describe the use of CP-µOCT for increasing the imaging contrast of microscopic birefringent crystals in fresh human coronary artery tissue *in situ* and validate the technology against histology with polarized light microscopy (PLM).

## 2 Materials and methods

The study was conducted under the approval of the Partners Institutional Review Board (IRB #2015P002522), and was performed in compliance with the Declaration of Helsinki.

### 2.1 CP-µOCT system

A description of the CP-µOCT instrument is provided in the Supplementary Materials. The capabilities of CP-µOCT to enhance the contrast of birefringent structures and to visualize synthetic microscopic crystals were tested (Supplementary Figures S1, S2).

### 2.2 Coronary sample preparation for CP-µOCT imaging

Whole fresh hearts were obtained from the National Disease Research Interchange (NDRI, Philadelphia, Pennsylvania). Hearts were acquired from patients who reported either a combination of gout and CAD (*n* = 10) or CAD without hyperuricemia and/or gout (*n* = 10). At the time of harvest, hearts were placed in phosphate buffered saline solution with antibiotics, packed on wet-ice and received at the Massachusetts General Hospital within 48 h postmortem. Prior to CP-µOCT imaging, the major coronary arteries were surgically dissected from the heart and opened longitudinally to expose the lumen. Three hearts (1 gout and 2 non-gout patients) were excluded due to severe calcification in all vessels or the presence of multiple stents. After thorough µOCT screening over the entire length of the coronary artery, 79 regions of interest (ROI) sized 1,000 (*x*) × 1,000 (*y*) × 400 (*z*) µm showing coronary atherosclerotic changes were selected for the study as previously described (9). The numbers of ROIs selected for the study were statistically comparable in gout patients and non-gout patients (5.6 ± 3.6 vs. 3.6 ± 2.3 ROIs per heart, *p* = 0.22).

### 2.3 Histology preparation

**Figure 1:**
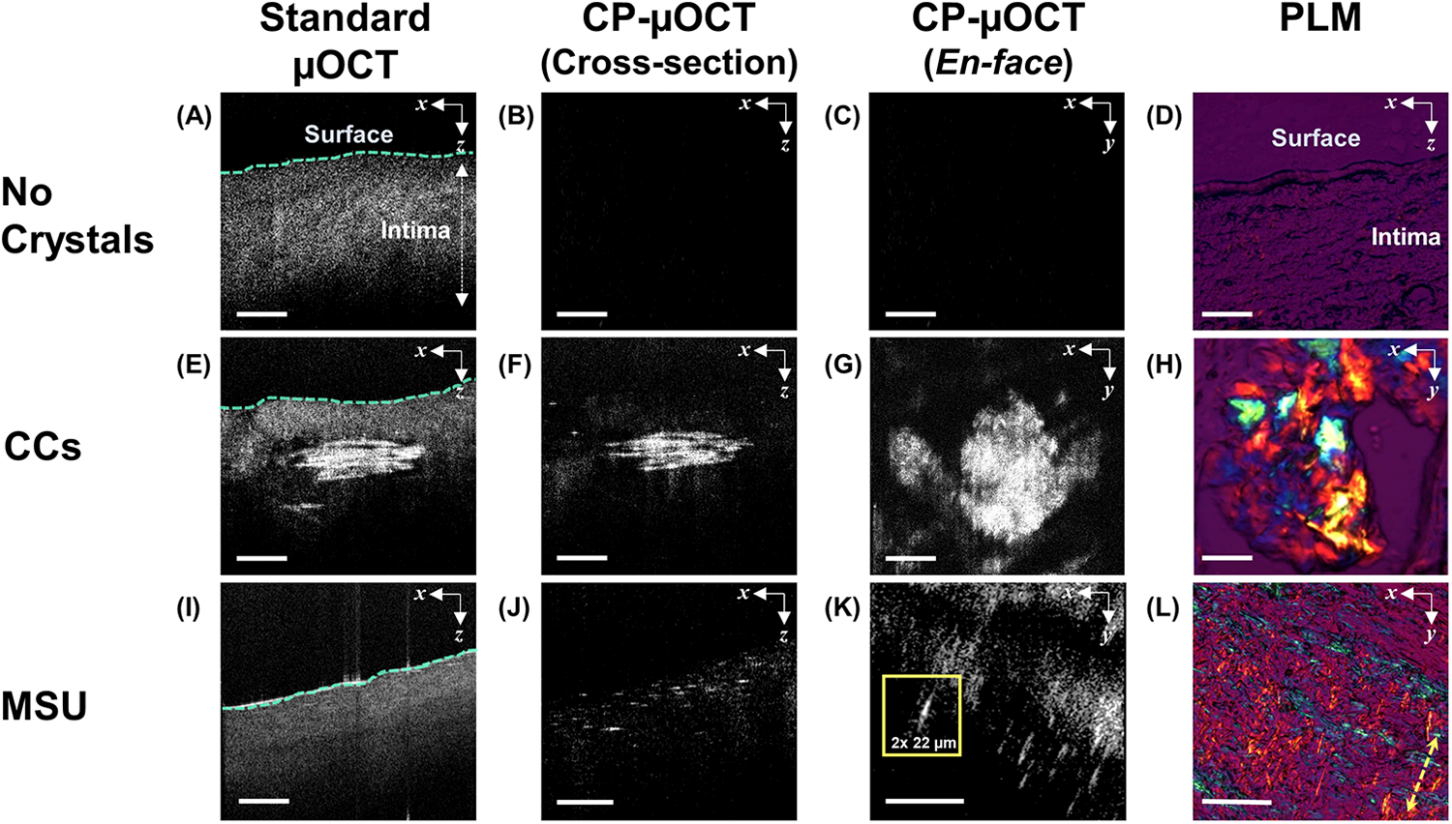
Histological validation of microscopic crystals identification from CP-µOCT images. **(A–D)** Standard µOCT, cross sectional and *en-face* CP-µOCT and PLM images of fresh healthy human cadaver coronary arteries with no crystalline structures, **(E–H)** with accumulated CCs and **(I–L)** with needle-shaped crystals suggestive of MSU. The *en-face* CP-µOCT image shows the difference between sheet-shaped and needle-shaped crystals corresponding to MSU and CCs, respectively **(G,K)**. The inset in **(K)** shows the size of an individual MSU crystal. Yellow double arrow indicates slow axis in PLM **(L)**. Scale bars, 100 µm **(A–D,I–L)**; 50 µm **(E–H)**. CC, cholesterol crystal; CP-µOCT, cross-polarized micro-optical coherence tomography; MSU, monosodium urate; PLM, polarized light microscopy.

Fiducial ink marks were applied to each corner of the ROI after µOCT imaging. Unfixed tissue from the ROI was then repeatedly cryo-sectioned (7 µm slide thickness) at 50 µm intervals for histology. To verify the orientation of crystals, several ROIs were selected for *en-face* sectioning (Figure 1). Unfixed tissue was then embedded in an optimal cutting temperature compound. Finally, unstained frozen slides were obtained for PLM; adjacent sections were submitted for hematoxylin and eosin (HE) staining. Unstained slides were imaged with a PLM (Eclipse E400 POL, Nikon, Japan) equipped with a first order retardation plate at 45° to the polarizer and an objective with a 40× magnification having a field of view of approximately 750 µm (*x*) × 400 µm (*z*). HE slides were digitized by a whole slide scanner (NanoZoomer, Hamamatsu Photonics, Shizuoka, Japan).

### 2.4 Synthesis of crystals

Methods are detailed in the Supplementary Materials. To test CP-µOCT's ability to detect the birefringent structures *in vitro*, the predominant biological crystalline of MSU and cholesterol monohydrate crystals were synthesized (Supplementary Figure S3).

### 2.5 Chemical study with uricase

**Figure 2:**
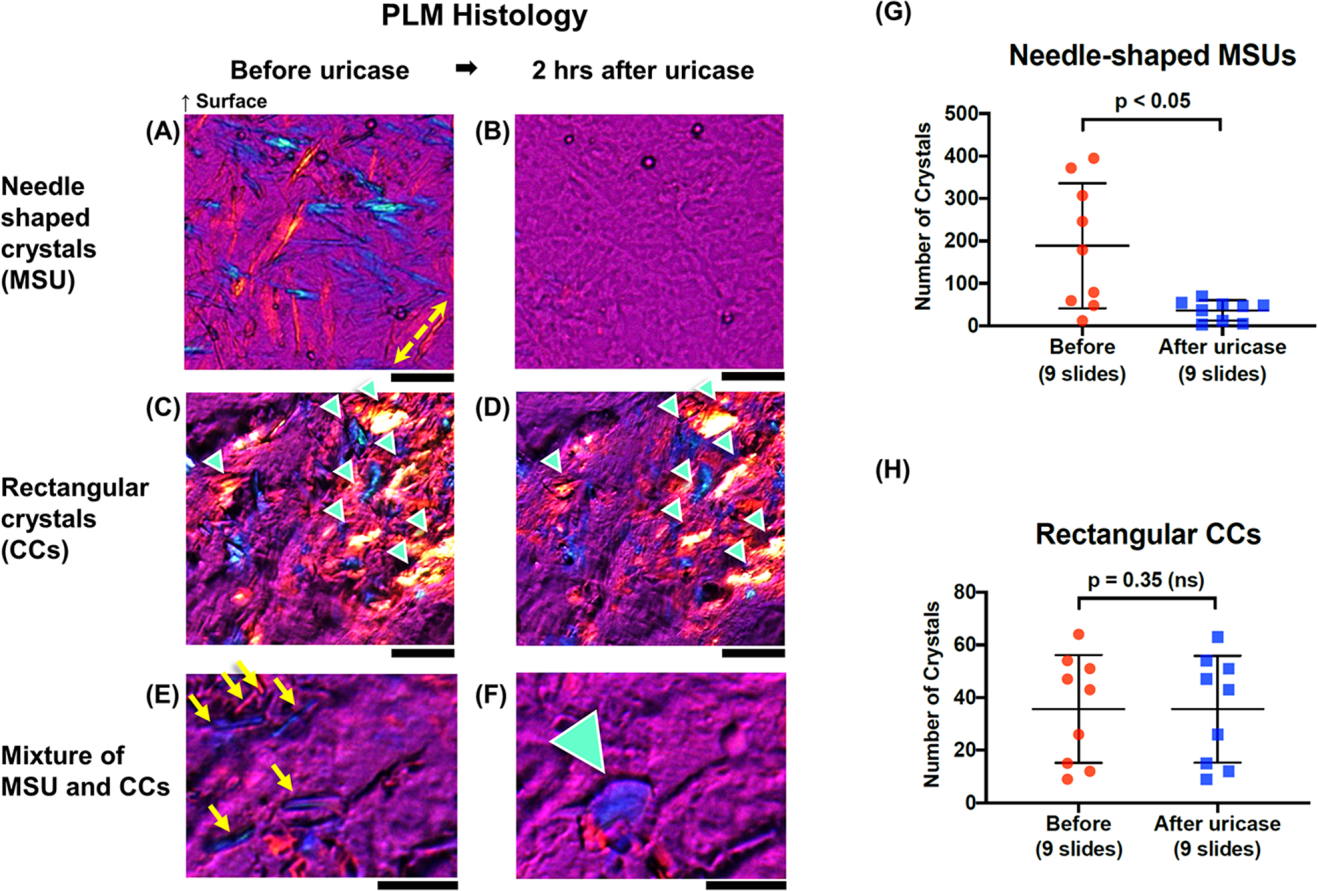
Chemical study with uricase. **(A,B)** PLM images of coronary arteries showing needle-shaped structures, **(C,D)** rectangular (sheet) shaped crystals (green arrowheads), and **(E,F)** a mixture of CC and MSU crystals before and 2 h after immersion in uricase. Slow axis in PLM is denoted by yellow double arrow **(A)**. Regions with mixed crystal types clearly showed that a rectangular crystal (green arrowhead in **F**) hidden by needle-shaped crystals (yellow arrows in **E**) appeared after immersion in uricase. Scale bars, 50 μm **(A–F)**. **(G,H)** Scatter dot plots showing counts of needle-shaped structures (MSU) and sheet shaped structures (CCs) before and after application of uricase. Error bars represent standard deviation around the mean. CC, cholesterol crystal; CP-μOCT, cross-polarized micro-optical coherence tomography; MSU, monosodium urate; PLM, polarized light microscopy.

To examine whether needle-shaped structures were MSU, 9 representative unstained slides were selected and immersed in uricase (U9375, Sigma-Aldrich, St. Louis, Missouri), an enzyme that specifically degrades uric acid to 5-hydroxylisourate, and incubated at 37℃ for 2 h (Figure 2).

### 2.6 Crystal count and size measurement on CP-µOCT

Cross-polarized images were analyzed using image analysis software ImageJ (National Institutes of Health, Bethesda, Maryland) (16). In this study, we have defined MSU crystals as a high-reflectivity birefringent structures having a length of ≤50 µm and thickness of ≤2.5 µm based on size measurements of isolated MSU crystals in synovial fluid (35 ± 15 µm × 1.9 ± 0.6 µm). CCs were defined as sheet-shaped structures with a length of greater than 50 µm. Crystal counts for MSU crystals were performed by generating a 3D-reconstructed CP-µOCT cross-sections, which were composed of 500 individual cross-sections (Supplementary Figure S4A). The 3D-reconstruction was further subdivided into 20 smaller 3D sub-reconstructions consisting of 25 cross-sectional images having a length of 50 µm (*y*), which is on the length scale exhibited by MSU crystals in synovial fluids, confirmed in a previous study (3). The CP-µOCT sub-reconstructions were cropped to match the PLM field of view [750 µm (*x*) × 400 µm (*z*)].

**Figure 3:**
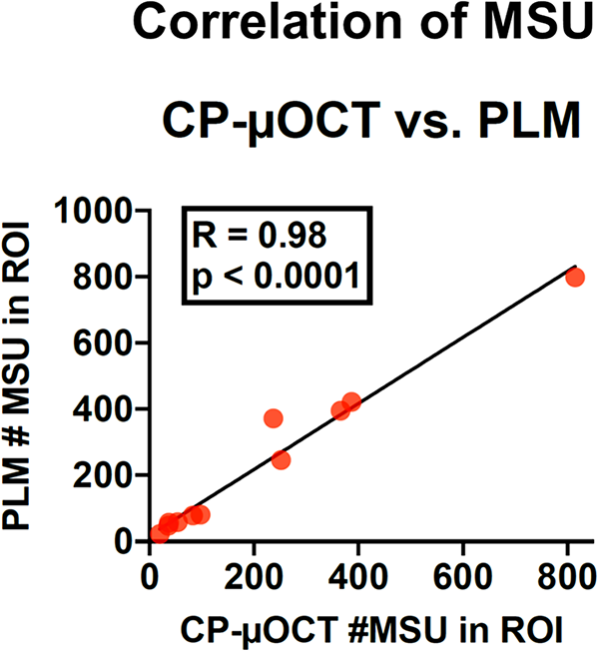
Correlation of MSU counted by CP-µOCT vs. PLM. Eleven CP-µOCT ROIs of 750 (*x*) × 1,000 (*y*) × 400 µm (*z*) and matching PLM slides (40× magnification, slides with 750 (*x*) × 400 µm (*z*) field of view) were chosen for the analysis. ROI, region of interest; other abbreviations as in Figure 1.

**Figure 4:**
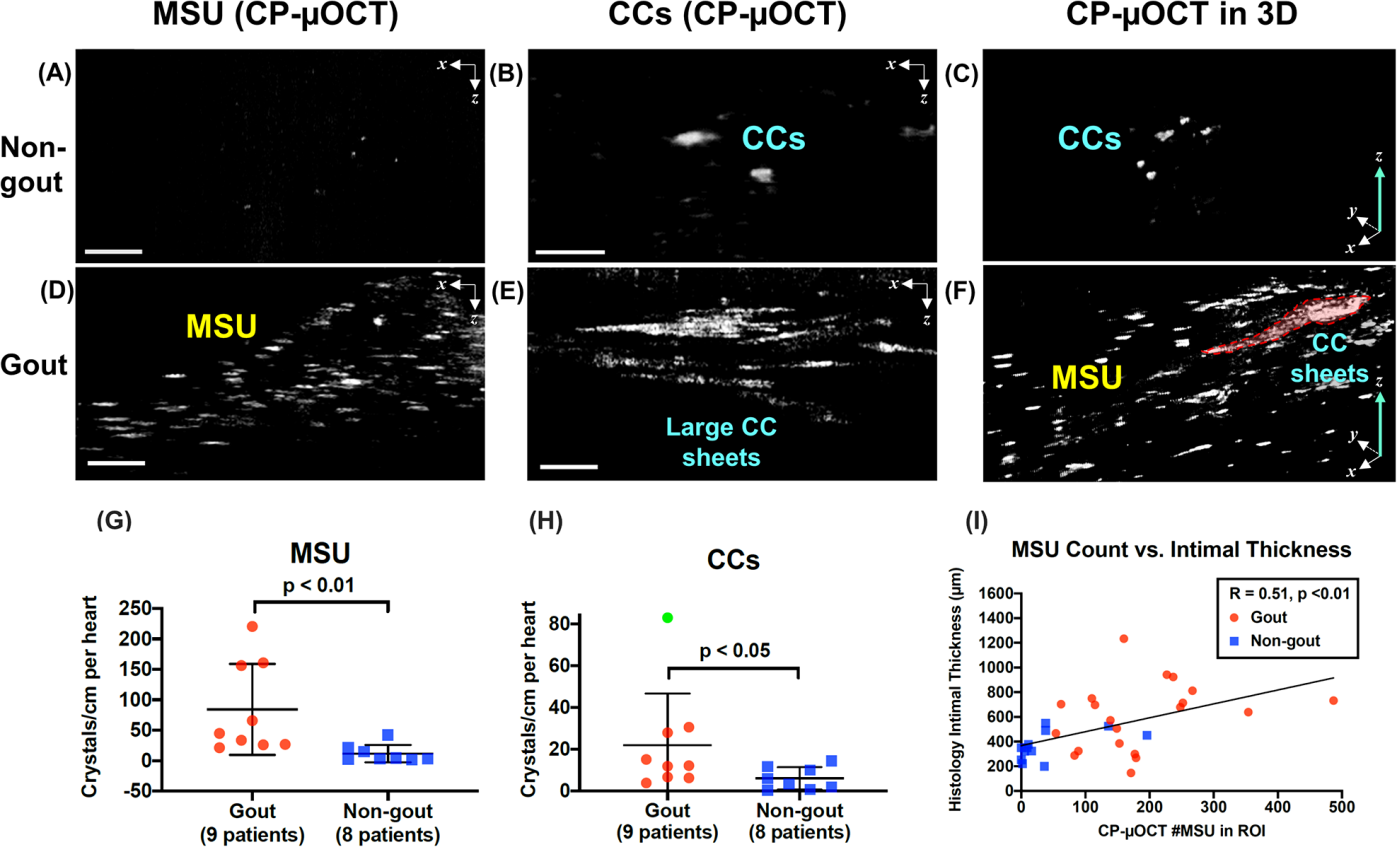
Comparison of crystal deposition in non-gout vs. gout patients. **(A–C)** Representative CP-µOCT images from a fresh human cadaver coronary artery of a non-gout patient and **(D–F)** from a gout patient showing that the gout patient has a higher number of coronary MSUs and CCs. **(G,H)** Scatter dot plots show that the number of MSU and CC crystals/cm was significantly higher in gout patients. Error bars represent standard deviation around the mean. An outlier (green dot) was removed from the analysis in **(H)**. **(I)** Correlation of the averaged maximum intimal thickness with the number of MSU (20 ROIs of gout patients and 12 ROIs of non-gout patients). Scale bars, 50 µm **(A,C,D,F)**; 100 µm **(B,E)**. Abbreviations as in Figures 1, 2.

MSU crystals were manually counted in each of the CP-µOCT sub-reconstructions and were correlated with MSU crystals counted within the field of view of corresponding PLM histology from the ROI (Figure 3). Crystals counts for all specimens were conducted independently by two investigators (KN and OOA). Intra-observer and inter-observer agreement of MSU crystal count on CP-µOCT was determined by calculating Lin's concordance correlation coefficient values [0.85 (95% CI: 0.60–0.95) and 0.85 (95% CI; 0.48–0.95), respectively]. The crystal count per artery was standardized by the total length of the artery (Figure 4 and Supplementary Figure S4A). The total arterial length per heart was comparable between gout and non-gout patients (21.8 ± 4.1 vs. 19.3 ± 4.8 cm, *p* = 0.14). Likewise, all crystals larger than 2.5 µm × 50 µm with sheet-shaped structures were counted as CCs. The size of the largest CC was measured in *en-face* CP-µOCT images and was compared between gout (*n* = 7) and non-gout patients (*n* = 5) (Supplementary Figure S4B).

### 2.7 Intimal thickness measurement and coronary tissue type categorization

To establish a relationship between prevalence of MSU in human coronary arteries and atherosclerotic burden, we compared MSU counts to the degree of intimal thickening in corresponding HE slides. Maximum intimal thickness was determined using NDP.view software (Hamamatsu Photonics, Japan), and was averaged over all slides from each ROI (Supplementary Figure S5). Maximum intimal thickness from CP-µOCT ROIs from gout (*n* = 20) and non-gout (*n* = 12) patients was chosen for the statistical analyses. Moreover, coronary tissue type from each slide was categorized as either intimal thickening (and/or xanthoma), pathological intimal thickening with lipid, fibroatheroma with necrotic core or calcific plaque as previously described (13) and compared between gout and non-gout patients (Supplementary Table S1).

### 2.8 Statistical analysis

Continuous variables were expressed as mean ± standard deviation. Unpaired student's *t*-test for normal distribution were used to analyze differences in continuous variables. Mann–Whitney *U*-test was used when not normally distributed. Chi-square test was used for categorical variables. A value of *p* < 0.05 was considered to be statistically significant. Correlations between continuous variables were analyzed using a linear regression model. The Lin's concordance correlation coefficient values for intra-observer and inter-observer agreement were calculated. The statistical analysis was performed with SPSS statistics 25 (IBM Corp, Armonk, New York) and R version 3.1.1 (http://www.r-project.org).

## 3 Results

### 3.1 Feasibility of CP-µOCT for imaging microscopic birefringent crystals

Side-by-side CP-µOCT images of a non-birefringent glass fragment and cholesterol monohydrate demonstrated the anticipated contrast enhancement for birefringent materials (Supplementary Figures S2A,B). Comparison of CP-µOCT and PLM images of MSU indicated that individual MSU crystals can be identified by the CP-µOCT system (Supplementary Figures S2C,D). When imaging a human cadaveric coronary plaque with µOCT, large aggregates of CC sheets (10, 11) were clearly visible above the tissue background (Supplementary Figure S2E). When CP-µOCT was used, the tissue background signal was reduced, resulting in a vast improvement in contrast that significantly increased the visibility of CC sheets and allowed smaller birefringent structures to become apparent (Supplementary Figure S2F).

### 3.2 Birefringent crystals visualization in human coronary plaques

To examine whether MSU and CCs could be seen by CP-µOCT in atherosclerotic plaque, we imaged fresh human cadaver coronary arteries in 3D (Figure 1). CP-µOCT demonstrated the absence of birefringent microstructures in minimally diseased arteries with intimal hyperplasia (Figures 1A–C), which was confirmed by corresponding PLM image (Figure 1D). In lipid-rich and necrotic regions, CC sheets (10, 11) were easily seen with standard µOCT (Figure 1E), and the lower background in CP-µOCT images significantly enhanced crystal contrast (Figures 1F,G), giving an appearance that was similar to that seen by PLM (Figure 1H). Although standard µOCT images showed no evidence of isolated needle-shaped crystals (Figure 1I), CP-µOCT-delineated individual needle-shaped crystals, were seen in cross-sectional and *en-face* CP-µOCT images (Figures 1J,K) and the corresponding PLM image (Figure 1L). The needle-shaped morphology of these micron-scale crystals was suggestive of MSU.

### 3.3 MSU crystals in human coronary plaques

We then examined the prevalence of MSU crystals in the coronary arteries of gout and non-gout patients (9 vs. 8 hearts) (Figure 4). While CP-µOCT identified few MSU crystals in non-gout patient coronaries (Figure 4A), gout patients exhibited a much higher number of coronary MSU crystals (Figure 4D). Statistical analysis showed that the number of MSU crystals in the coronaries of gout patients was higher than that in non-gout patients (84 ± 75 vs. 12 ± 14 crystals/cm in coronary arteries per heart, *p* < 0.01) (Figure 4G). Even though plaque type distribution determined by histology was statistically equivalent in the two groups (Supplementary Table S1), intimal thickness was significantly greater in gout patients vs. non-gout patients (604 ± 272 vs. 367 ± 116 µm, *p* < 0.05) (Supplementary Figure S5B). A moderate-strong correlation existed between intimal thickness and number of CP-µOCT delineated coronary MSU (R = 0.51, *p* < 0.01) (Figure 4I).

### 3.4 CCs in human coronary plaques

Gout patients had an increase in the number of CP-µOCT-delineated coronary CCs (22 ± 25 vs. 6 ± 6 crystals/cm in coronary arteries per heart, *p* < 0.05) (Figure 4H). In addition, large CC sheets were found in abundance in the coronaries of gout patients (Figure 4E), whereas smaller crystal accumulations were seen in non-gout patients (Figure 4B). The size of CCs was significantly larger in the coronaries of gout patients than in non-gout patients (22,377 ± 16,543 vs. 1,798 ± 406 µm^2^, *p* < 0.01). 3D reconstructed CP-µOCT images showed that coronary CCs were surrounded by a large number of MSUs in gout patients (Figure 4F) while smaller CCs were usually isolated in non-gout patients (Figure 4C). Indeed, a moderate-strong correlation was found between MSU and CC counts in coronary plaque (*R* = 0.62, *p* < 0.01). A very strong correlation was found between intimal thickness and the size of coronary CCs segmented by CP-µOCT (*R* = 0.81, *p* < 0.01).

## 4 Discussion

The major findings of the present study are that (1) CP-*μ*OCT enabled the discrimination of birefringent microscopic crystals (urate, cholesterol) in human cadaver coronary plaque based on crystal morphology made evident by increased contrast, (2) there was a significant elevation in the number of CP-µOCT-delineated MSU crystals in patients who had a history of gout compared to patients without gout, (3) there was a moderate-strong correlation between CP-µOCT-delineated MSU and intimal thickness, (4) there was a moderate-strong correlation between MSU and CC crystal counts, and (5) there was a very strong correlation between CC burden and intimal thickness.

### 4.1 Microscopic birefringent crystals in CAD

CCs are known as birefringent crystalline components that are commonly noted in coronary atherosclerotic plaques (1). Evidence has accumulated for CC visualization by commercially available conventional OCT (17, 18). More recently, we have reported the feasibility of µOCT for visualization of layered CC sheets in human cadaver coronary arteries (10–12). Relatively little is known about the existence and extent of MSU, another form of birefringent crystals, in human coronary plaque due to the lack of a methodology for imaging these subcellular structures in atherosclerotic lesions *in situ*. MSU crystals are much smaller than CCs and are therefore not capable of being resolved by conventional OCT. As standard fixatives and solvents (e.g., formalin) and standard graded ethanol concentrations used in histopathology processing can fully dissolve crystals (19, 20), it is important to have an imaging technology that can visualize microscopic crystals in fresh, unfixed human specimens. Here, we demonstrate CP-μOCT for identification and quantification of individual MSU crystals in fresh human cadaver coronary plaque.

PLM has been a gold standard technique for histologic visualization of MSUs, as the crystals alter the polarization of incident light through their intrinsic birefringence properties (21). One of the few PLM studies on MSU quantification in human explanted coronary arteries reported that 6 coronary arteries out of 55 alcohol-fixed hearts explanted from heart transplant patients harbored negative birefringent crystals suggestive of MSU (22). However, the study showed only a few PLM images of isolated microtophus with negative birefringence and did not distinguish MSU-like structures from other crystals such as CCs. Evaluation of MSU burden in tissue with PLM requires serial frozen sections and high microscopic magnification, which is time consuming and destructive to the sample. The orientation of the crystals may be important in understanding their distribution and formation. CP-µOCT is superior to single-section histology slides as this technology maintains 3D orientation of the birefringent crystals *in situ*.

A recent study reported that dual-energy computed tomography (DECT) is capable of identifying MSU distribution in human aortic and coronary arteries (23). DECT has many advantages for studying coronary MSU deposits, especially its clinical availability and non-invasive assessment. Yet, DECT cannot precisely identify individual crystals because its spatial resolution is insufficient. Thus, its accuracy for MSU visualization compared to co-registered histology remains equivocal. It is also critical to inform pathogenesis based on crystal distributions in their microscopic morphologic context. The majority of coronary plaques imaged by CP-µOCT was not histologically advanced (Supplementary Table S1), suggesting that CP-µOCT allows the precise quantification of MSU deposition by counting individual crystals even in the early stages of coronary atherosclerosis. Owing to its high spatial resolution, CP-µOCT will make it possible to correlate the extent of birefringent crystal deposition with other key plaque components such as CCs and macrophages (10, 11). These advantages of CP-µOCT may be essential for elucidating the underlying mechanisms of CAD in gout or hyperuricemic patients.

### 4.2 Clinical implications

CCs have long been identified as a possible therapeutic target for CAD because these crystals can be a trigger of inflammasome-mediated cytokine production/activation (1). Furthermore, CCs can tear membrane and trigger plaque disruption (24). A recent OCT study reported that CCs are more abundant in plaques that have ruptured, potentially supporting this mechanism of plaque rupture (18).

Likewise, MSU has shown to induce gouty inflammation through NLRP3 inflammasome activation, and interleukin-1β secretion (6). Although it has been widely accepted that hyperuricemia/gout is associated with CAD (25), the effect of urate-lowering therapy on CAD is still unclear (26).

Imaging cadaver coronary arteries obtained from CAD patients, we found that there was a significant increase in the number of CP-µOCT-delineated MSU crystals in patients who have a history of gout than in patients without gout. Furthermore, a positive correlation was found between CP-µOCT-delineated MSU and intimal thickness, supporting the hypothesis in part that gout/hyperuricemia promotes the advancement of CAD (25). Interestingly, a similar increase in the number of CCs was found in gout patients and the CC burden was also correlated to intimal thickness. Co-crystallization of CCs and calcium phosphate crystals has been previously reported (27). A similar association may exist in between CCs and MSU, although this phenomenon remains to be fully validated in cardiovascular system (28). Environmental factors, such as local temperature and pH level, are involved in the crystal formation. Local inflammatory levels exacerbated by crystal formation (6, 7) may influence environmental factors and may also accelerate another type of crystal formation. Determining whether MSUs as well as CCs are a driver of atherosclerosis resulting in increased plaque burden should be the topic of future CP-μOCT investigations.

Most recently, colchicine for acute gouty flare has been proposed as anti-inflammatory strategy for secondary prevention in CAD patients (7–9). Colchicine was found to block the NLRP3 inflammasome that can be triggered by MSUs (6) and CCs (7) as previously mentioned. μOCT is capable of typing leukocytes and imaging pseudopods that inform on the activity of inflammatory cells (10–12). CP-µOCT may enable the visualization of inflammatory cells associated with birefringent crystalline components in human coronary plaques, and thus this technology could be used for understanding how novel anti-inflammatory agents affect coronary atherosclerotic plaque progression.

### 4.3 Study limitations

This was an *ex vivo* study, and thus CP-µOCT's feasibility for imaging MSU crystals should be verified *in vivo*. With the recent development of a µOCT coronary catheter (12), future studies can be envisioned to examine the role of coronary MSUs in the pathogenesis of CAD *in vivo*. The majority of coronary tissue types was intimal thickening/xanthoma and pathological intimal thickening (Supplementary Table S1); future studies should be conducted to verify these results in more advanced lesions. The depth range of CP-µOCT is approximately 300 µm, which may not be sufficient for a full detection of birefringent materials over the entire human coronary arterial wall. No patient history regarding serum uric acid levels, presence of gout in synovial fluids, treatment history of hyperuricia/gout and cause of death was available. For crystal counts on the CP-µOCT images, CCs were defined as birefringent crystals with lengths > 50 µm, and so it is unclear whether our method differentiates small CC's from MSUs.

## 5 Conclusions

The present results demonstrate a significant increase in CP-µOCT delineated crystals in gout vs. non-gout patients, suggesting that this technology can be used to improve our understanding of crystal-driven coronary pathogenesis and potentially monitor response to crystal abrogating therapy.

## Data Availability

The raw data supporting the conclusions of this article will be made available by the authors upon reasonable request.
