# Supplementary material for: Imaging human coronary cholesterol/urate crystals with cross-polarized micro-optical coherence tomography
Source: Front Cardiovasc Med. 2024 Oct 28;11:1433227. doi: 10.3389/fcvm.2024.1433227 (PMC11551715; doi:10.3389/fcvm.2024.1433227)
Supplement: Supplementary file 1 [file Datasheet1.pdf]

Supplementary Materials

Imaging Human Coronary Cholesterol/Urate Crystals with Cross-Polarized Micro-Optical Coherence Tomography

Kensuke Nishimiya (KN), M.D., Ph.D.;[1] Gargi Sharma (GS), Ph.D.;[1] Kanwarpal Singh (KS), Ph.D.;[1] Osman Oguz Ahsen (OOA).;[1] Joseph A. Gardecki (JAG), Ph.D.;[1] *Guillermo J. Tearney (GJT), M.D., Ph.D.[1,2,3]

1. Wellman center for photomedicine, Harvard Medical School and Massachusetts General Hospital, Boston, Massachusetts, USA
2. Department of Cardiovascular Medicine, Tohoku University Graduate School of Medicine, Sendai, Miyagi, Japan
3. Harvard-MIT Division of Health Sciences and Technology, Cambridge, Massachusetts, USA
4. Department of Pathology, Harvard Medical School and Massachusetts General Hospital, Boston, Massachusetts, USA

*Correspondence: Guillermo J. Tearney, MD, PhD, FACC, FACP, gtearney@mgb.org

Total word count: 1,351 words with 1 table, 5 supplementary figures and 3 references

Contents:

Supplementary Methods for CP-μOCT System ···················· *Pages 2-3*

Supplementary Methods for Synthesis of Crystals ······ ········· *Page 3*

References ·· *Page 4*

Supplementary Table 1 ·· *Page 5*

Supplementary Figure legends 1-5 ········ ···················· *Pages* 6-11

Supplementary Methods

Cross-Polarized Micro-Optical Coherence Tomography (CP-μOCT) System

The experimental schematic for the CP-μOCT system is shown in **Supplementary Figure 1A**. This system builds on our prior μOCT systems and enables acquisition of co- and cross-polarized images. Broadband unpolarized light from a super continuum laser (NKT Photonics, Denmark) was linearly polarized using a broadband linear polarizer. The linearly polarized light was coupled to a single-mode fiber and collimated using an achromatic lens (L2). Polarization controllers were used to maintain the linear polarization of the light while traveling through the single-mode fiber. The central portion of the collimated light was reflected using a 45-degree angled rod mirror beam splitter (RM-BS, Edmund optics, USA). The transmitted light was used to illuminate the sample. An achromatic quarter wave plate (QWP, B. Halle, Germany) was placed in the reference arm as well as sample arm to control the polarization of the light. The reference signal after the QWP was focused using an achromatic lens (L3) onto a reference mirror which was placed on translational stage. The reference arm QWP was rotated 0 degree and 45 degrees with respect to the sample arm QWP to acquire co-and cross-polarized images, respectively. The sample arm QWP was aligned to maintain linear polarization. Linearly polarized light was focused on the sample using an achromatic lens (L4) and scanned over the sample using two-dimensional galvanometer mirrors. Annular apodization due to the RM-BS extended the depth of field to ~200 μm, preserving transverse resolution over longer imaging depths (1). Reflected reference and sample light were combined at the RM-BS and sent to a custom spectrometer that recorded the interference signal. The spectrometer consisted of a telescope lens (L5, L6), and a 10 μm pinhole, a mirror, a 900 lines per millimeter volume holographic grating (Wasatch Photonics, USA), a camera lens (Nikon, Japan) and a 4,096 pixel line scan camera (Basler, Germany)

operated at 20 kHz spectra per second. The axial and lateral resolution of the μOCT system was measured to be 1.8 μm and 2.2 μm in air, respectively and the field of view was 1 x 1 mm. The sensitivity of the system was measured to be 90 dB.

To test the polarization performance of the CP-μOCT system, we used a mirror as the sample, which preserves the polarization state of the linearly polarized incident light. The reference arm QWP was rotated between -45 and +45 degrees with respect to the sample arm QWP, corresponding to a change of retardance from –90 to +90 degrees in the double pass configuration. The change in polarization was calculated from the amplitude of the co-polarized (A_{co}) and cross- polarized (A_{cross}) images using Equation (1) and is plotted in **Supplementary Figure 1B**. The measured retardance angle (δ) values matched closely to the rotational angles of the QWP with an R of 1.00 ($p<0.01$).

$$\delta = \tan^{-1}\left[\frac{A_{cross}}{A_{co}}\right] \quad (1)$$

Synthesis of Crystals

To test the CP-μOCT's ability to detect the birefringent structures *in vitro*, the predominant biological crystalline of monosodium urate (MSU) and cholesterol monohydrate crystals were synthesized (**Supplementary Figure 3**). MSU crystals were synthesized by adding sodium hydroxide to uric acid (Sigma-Aldrich, Sigma-Aldrich, St. Louis, Missouri) dissolved in water (2). The solution was boiled and allowed to cool slowly. Needle-shaped MSU were formed and washed with water. The cholesterol monohydrate crystals were synthesized by recrystallizing cholesterol (Sigma-Aldrich, Sigma-Aldrich, St. Louis, Missouri) in an ethanol-water (95:5) solution with controlled cooling followed by washing with distilled water (3).

References

1. de Boer JF, Hitzenberger CK, Yasuno Y. Polarization sensitive optical coherence tomography - a review. ***Biomed. Opt. Express.*** 2017;8:1838-73. https://doi.org/10.1364/BOE.8.001838.
2. Seegmiller JE, Howell RR, Malawista SE. The inflammatory reaction to sodium urate: its possible relationship to the genesis of acute gouty arthritis. ***JAMA*** 1962;180:469-75.
3. Loomis CR, Shipley GG, Small DM. The phase behavior of hydrated cholesterol. ***J. Lipid. Res.*** 1979;20:525-35.

Supplementary Table

Supplementary Table 1. Coronary tissue type categorization

	Gout	Non-gout	P value
Histology, n	361	297	
Tissue category			
Intimal thickening/Xanthoma, n (%)	95 (26)	91 (31)	0.08
Pathological intimal thickening, n (%)	195 (54)	142 (48)	0.11
Fibroatheroma, n (%)	6 (2)	2 (1)	0.25
Calcific plaque, n (%)	71 (20)	62 (21)	0.70

Categorical variables are expressed as n (%). The tissue type of each hematoxylin and eosin slide was categorized as intimal thickening (and/or xanthoma), pathological intimal thickening with lipid-pool, fibroatheroma with necrotic core and calcific plaque. Several slides overlapped calcific plaques and other tissue types. Plaque distribution was equivalent in the two groups.

Supplementary Figures

Supplementary Figure 1. Experimental Set-Up

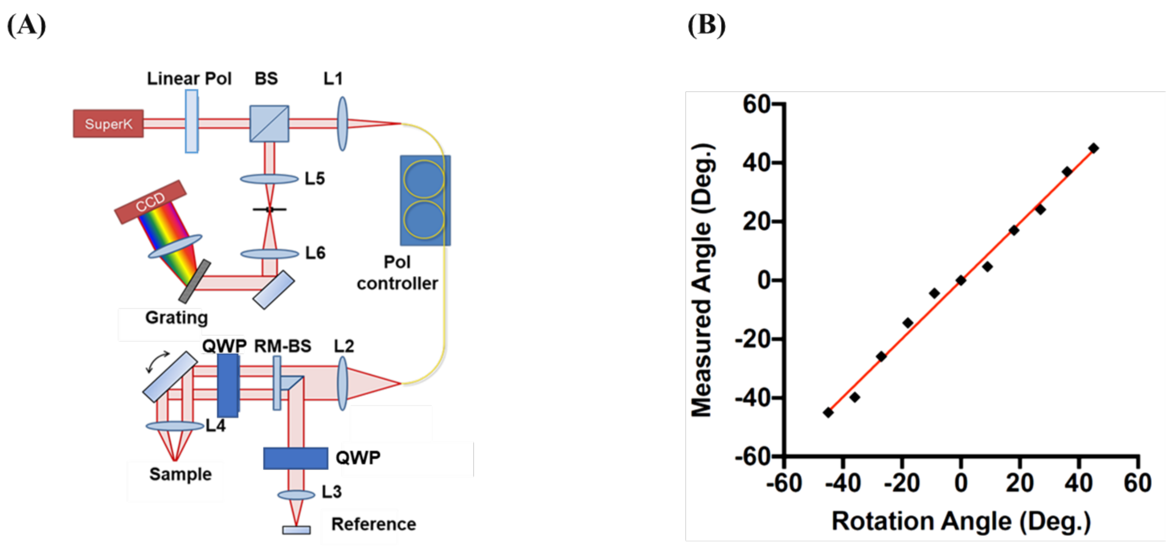

(**A**) Experimental schematic of the CP-µOCT system. (**B**) Polarization measurement verification by rotating the QWP.

BS=beam splitter; CCD=charge coupled device; CP-µOCT=cross-polarized micro optical coherence tomography; QWP=quarter wave plate; RM-BS=rod mirror beam splitter.

Supplementary Figure 2. CP-µOCT System Verification

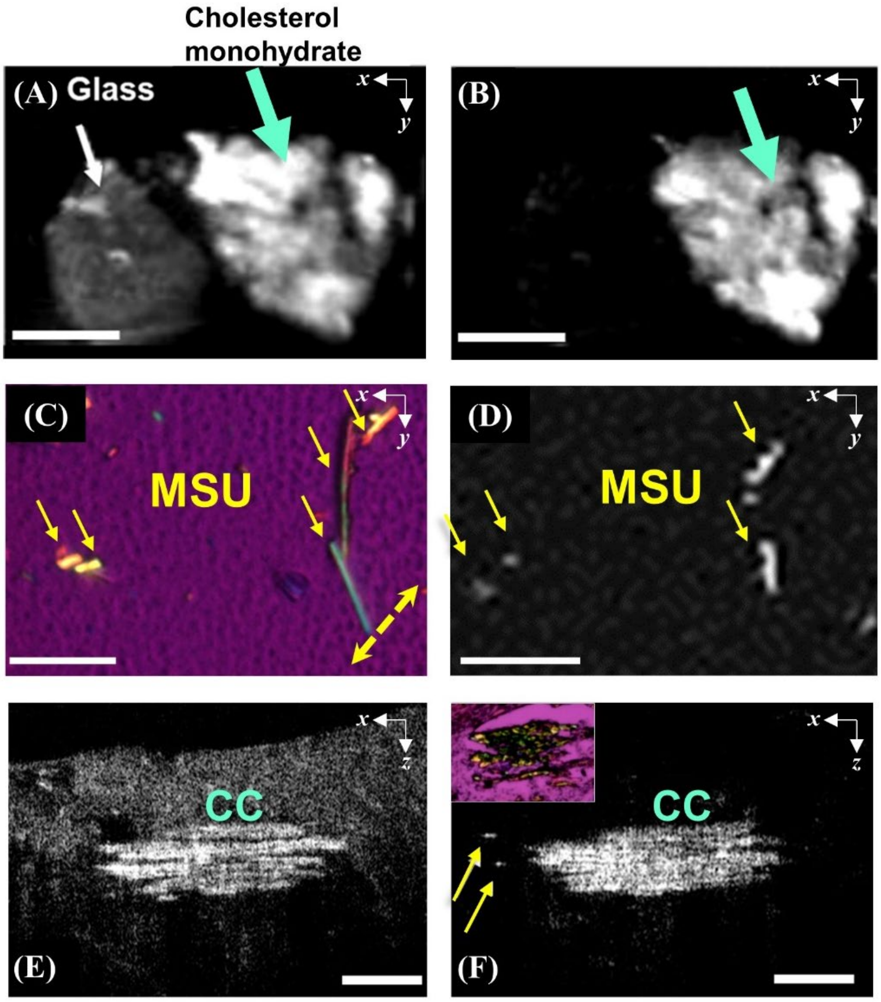

(**A**) Glass and synthesized cholesterol monohydrate crystal (green arrow) imaged with standard µOCT and (**B**) with *en face* CP-µOCT. (**C**) MSU observed with PLM (yellow arrows) show the anticipated negative birefringence relative to the slow axis in PLM (denoted by yellow double arrow) (**D**) which appear similar in the *en face* CP-µOCT image. (**E**) CC sheets measured in the coronary artery using standard cross-sectional µOCT and (**F**) CP-µOCT image, with yellow arrows indicating needle-shaped crystals.

Scale bar for (c, d, g, h), 50 µm; for (e, f), 20 µm.

CC=cholesterol crystal; MSU=monosodium urate; PLM=polarized light microscopy.

Supplementary Figure 3. Chemical Study with Uricase

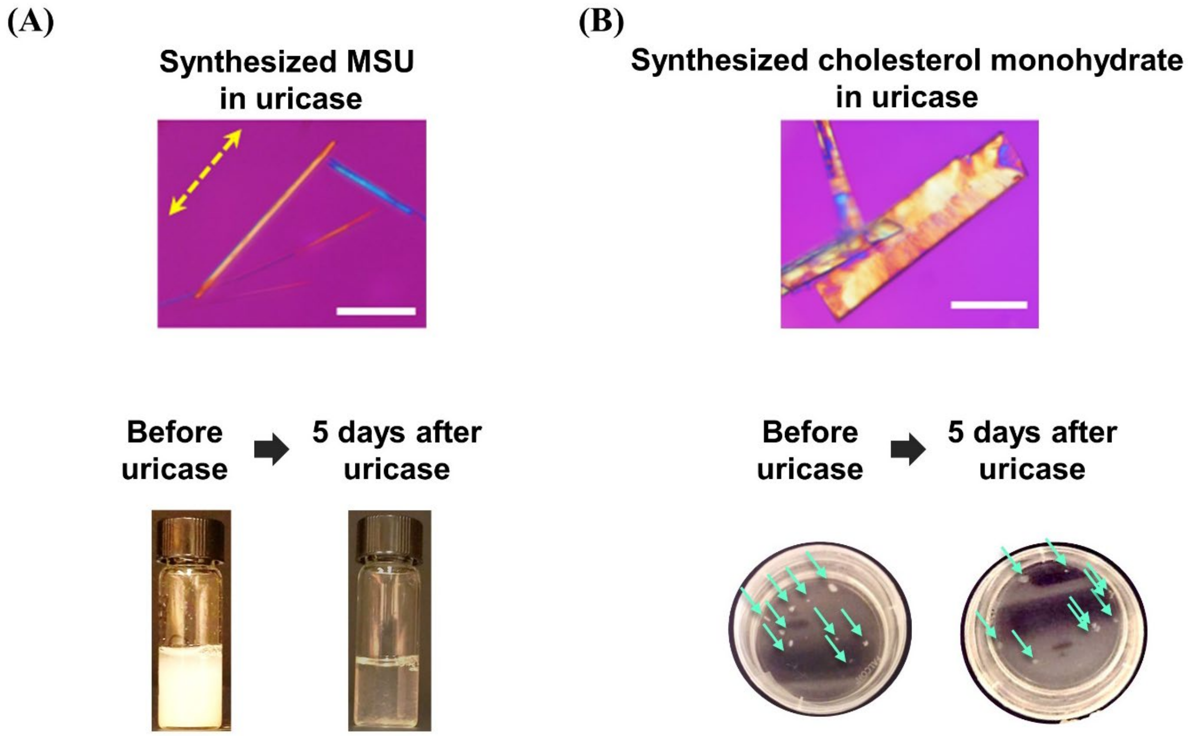

(**A**) Results showing synthesized MSU and (**B**) cholesterol crystals immersed in uricase before and after 5 days.

Scale bars, 25 μm (**A, B**). Abbreviations as in **Supplementary Figure 2**.

Supplementary Figure 4A Crystal Count and Size Measurement on CP-µOCT

(A) Crystal count by CP-µOCT

CP-µOCT dataset of a ROI (with 500 cross-sections)

Divide into 20 smaller 3D sub-reconstructions
(each with 25 cross-sections)

Count crystals manually in 3D-subreconstructions

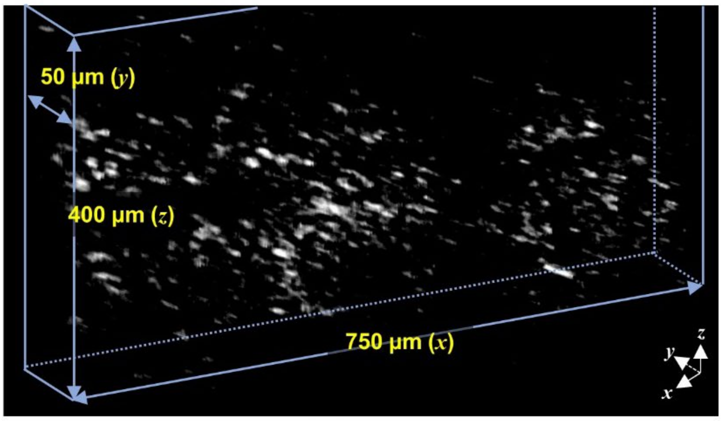

Normalize total crystal count by the total length of the coronary artery
(Unit = **crystals/cm per heart**).

(**A**) A CP-µOCT dataset of a ROI with 500 cross-sections was divided into 20 smaller datasets, each with 25 cross-sections. CP-µOCT cross-sections of the smaller datasets were then reconstructed in 3D and were cropped at 750 (*x*) x 50 (*y*) x 400 (*z*) µm to match the 750 x 400 µm PLM field of view. MSU (≤2.5 x 50 µm) and larger sized CCs (>2.5 x 50 µm) were manually counted on the 3D reconstructed CP-µOCT. Total crystal counts in ROIs per heart were normalized by the total length of the coronary artery (unit = crystals per cm per heart).

Supplementary Figure 4B Crystal Count and Size Measurement on CP-μOCT

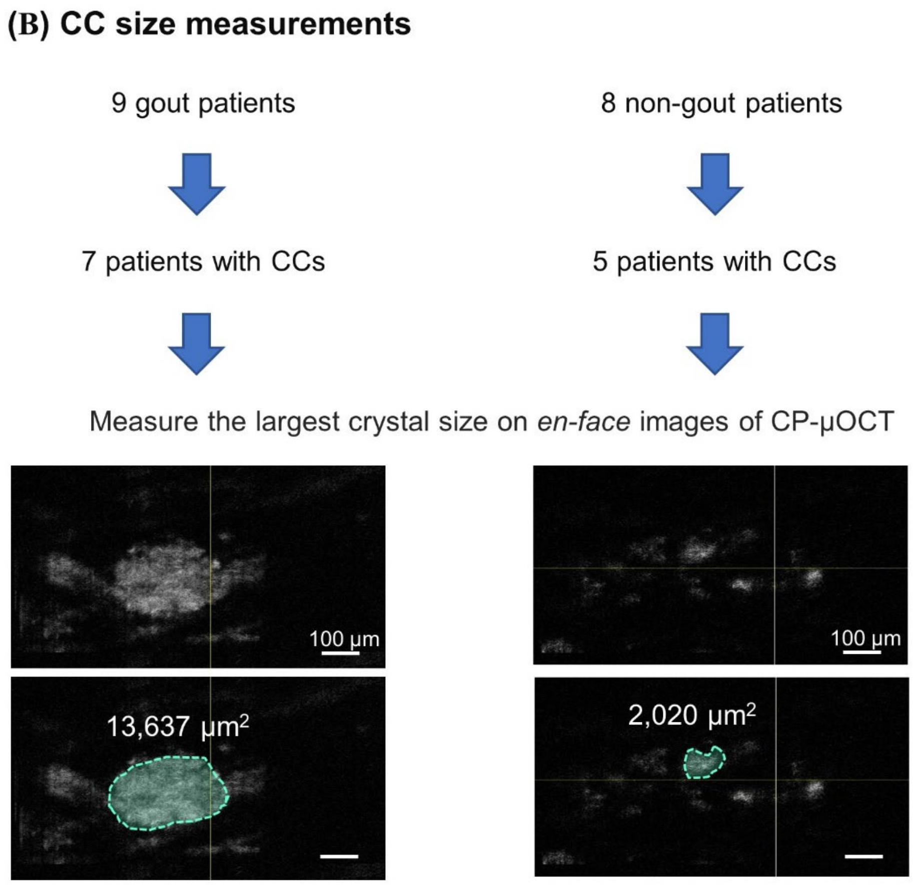

(**B**) The size of the largest CC was measured on *en-face* CP-μOCT images and was compared between gout vs. non-gout patients.

ROI=region of interest; other abbreviations as in **Supplementary Figure 2**.

Supplementary Figure 5 Histological Analysis of Human Coronary Plaque

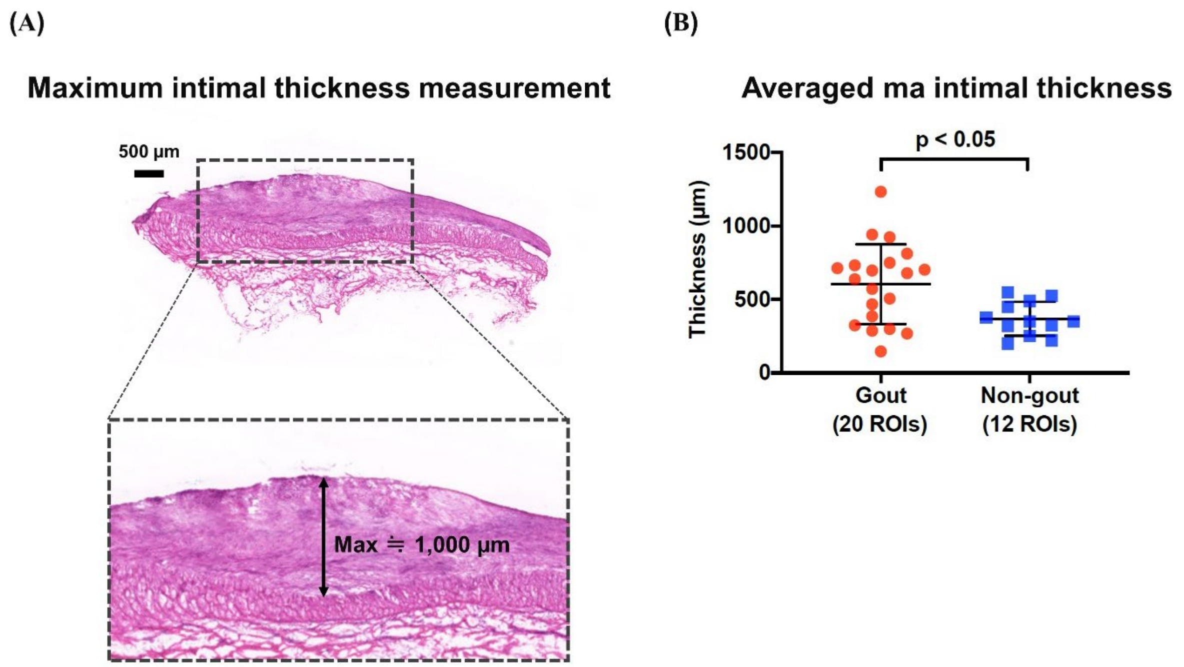

(**A**) Maximum intimal thickness was determined using a H&E stained slide and averaged in each dataset. (**B**) Scatter dot plots showing averaged intimal thickening of gout vs. non-gout patients. Error bars represent standard deviation around the mean.

Abbreviations as in **Supplementary Figure 5**.
